# Performance data of CH_3_NH_3_PbI_3_ inverted planar perovskite solar cells via ammonium halide additives

**DOI:** 10.1016/j.dib.2019.104817

**Published:** 2019-11-15

**Authors:** Muhammad Jahandar, Nasir Khan, Muhammad Jahankhan, Chang Eun Song, Hang Ken Lee, Sang Kyu Lee, Won Suk Shin, Jong-Cheol Lee, Sang Hyuk Im, Sang-Jin Moon

**Affiliations:** a Advanced Materials Division, Korea Research Institute of Chemical Technology (KRICT), 141 Gajeong-ro, Yuseong, Daejeon 34114, Republic of Korea; b Advanced Materials and Chemical Engineering, University of Science and Technology (UST), 217 Gajeongro, Yuseong, Daejeon 34113, Republic of Korea; c Energy Materials Research Center, Korea Research Institute of Chemical Technology (KRICT), 141 Gajeong-ro, Yuseong, Daejeon 34114, Republic of Korea; d Department of Chemical and Biological Engineering, Korea University, 145 Anam-ro, Seongbuk-gu, Seoul 136-713, Republic of Korea

**Keywords:** CH_3_NH_3_PbI_3_ perovskite, Inverted planar structure, Ammonium halide additives, anti-solvent engineering, Perovskite grain size

## Abstract

The data provided in this data set is the study of organic-inorganic hybrid perovskite solar cells fabricated through incorporating the small amounts of ammonium halide NH_4_X (X = F, Cl, Br, I) additives into a CH_3_NH_3_PbI_3_ (MAPbI_3_) perovskite solution and is published as “High-Performance CH_3_NH_3_PbI_3_ Inverted Planar Perovskite Solar Cells via Ammonium Halide Additives”, available in Journal of Industrial and Engineering Chemistry [1]. A compact and uniform perovskite absorber layer with large perovskite crystalline grains, is realized by simply incorporating small amounts of additives into precursor solutions, and utilizing the anti-solvent engineering technique to control the nucleation and growth of perovskite crystal, turning out the enhanced device efficiency (NH_4_F: 14.88 ± 0.33%, NH_4_Cl: 16.63 ± 0.21%, NH_4_Br: 16.64 ± 0.35%, and NH_4_I: 17.28 ± 0.15%) compared to that of a reference MAPbI_3_ device (Ref.: 12.95 ± 0.48%). In addition, this simple technique of ammonium halide addition to precursor solutions increase the device reproducibility as well as long term stability.

**Table:** Specifications Table

Subject	Materials Science
Specific subject area	Solar Energy conversion to electricity, Perovskite Photovoltaics.
Type of data	Graph Figure
How data were acquired	UV-2550 UV-Vis spectrophotometer, SEM, XRD, Polaronix K201 Solar Simulator, K3100 Spectral IPCE Measurement System, KEITHLEY 236 Source Measure Unit, GIWAXS, etc. OriginPro 8.5
Data format	Raw and Analysed
Parameters for data collection	GIWAXS study, JV characteristic curves, Light Intensity dependant behaviour of JV curves, JV scan speed, forward and reverse scan.
Description of data collection	All Data has been gathered in accordance to standard conditions
Data source location	Korea Research Institute for Chemical Technology (KRICT), Daejeon/Yuseong gu/Jang dong South Korea 36.3881° N, 127.3603° E
Data accessibility	With the article
Related research article	Muhammad Jahandar, Nasir Khan, Hang Ken Lee, Sang Kyu Lee, Won Suk Shin, Jong-Cheol Lee, Chang Eun Song, and Sang-Jin Moon “High-Performance CH3NH3PbI3-Inverted Planar Perovskite Solar Cells with Fill Factor Over 83% via Excess Organic/Inorganic Halide” ACS Appl. Mater. Interfaces DOI: 10.1021/acsami.7b11083 Muhammad Jahandar, Nasir Khan, Muhammad Jahankhan, Chang Eun Song, Hang Ken Lee, Sang Kyu Lee, Won Suk Shin, Jong-Cheol Lee, Won-Wook So, Sang Hyuk Im, and Sang-Jin Moon “High-Performance CH_3_NH_3_PbI_3_ Inverted Planar Perovskite Solar Cells via Ammonium Halide Additives” J. Ind. Eng. Chem. 80 (2019) 265–272. https://doi.org/10.1016/j.jiec.2019.08.004

**Table:** 

Valve of Data • This data describes the effects of ammonium halide (i.e. NH_4_F, NH_4_Cl, NH_4_Br, NH_4_I) additives with varying concentration on the nucleation and crystallization of perovskite film formation with respect to the reference MAPbI_3_ film. This result can draw the other perovskite photovoltaic researchers to design and fabricate stable and reproducible devices. • This data compares the photovoltaic performance of the reference MAPbI_3_ with the modified MAPbI_3_+NH_4_X (X = F, Cl, Br, I) with different molar concentration of NH_4_X. • This data shows the enhanced nucleation and controlled crystal growth with respect to the reference MAPbI_3_ film. • This data describes the state-of-the-art and facile technique for better reproducibility and stability of the perovskite solar cells.

## 1 Data

**Fig. 1:**
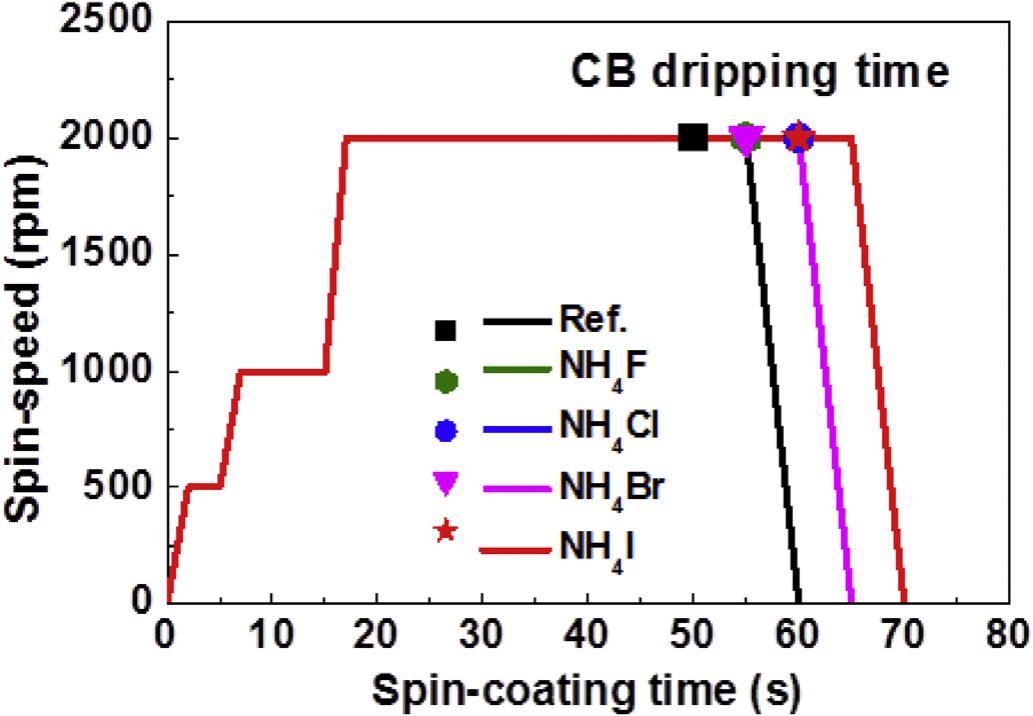
Schematic diagram of spin-coating process from perovskite precursor solution with CB dripping time for reference and NH_4_X (X = F, Cl, Br, I) incorporating perovskite films.

**Fig. 2:**
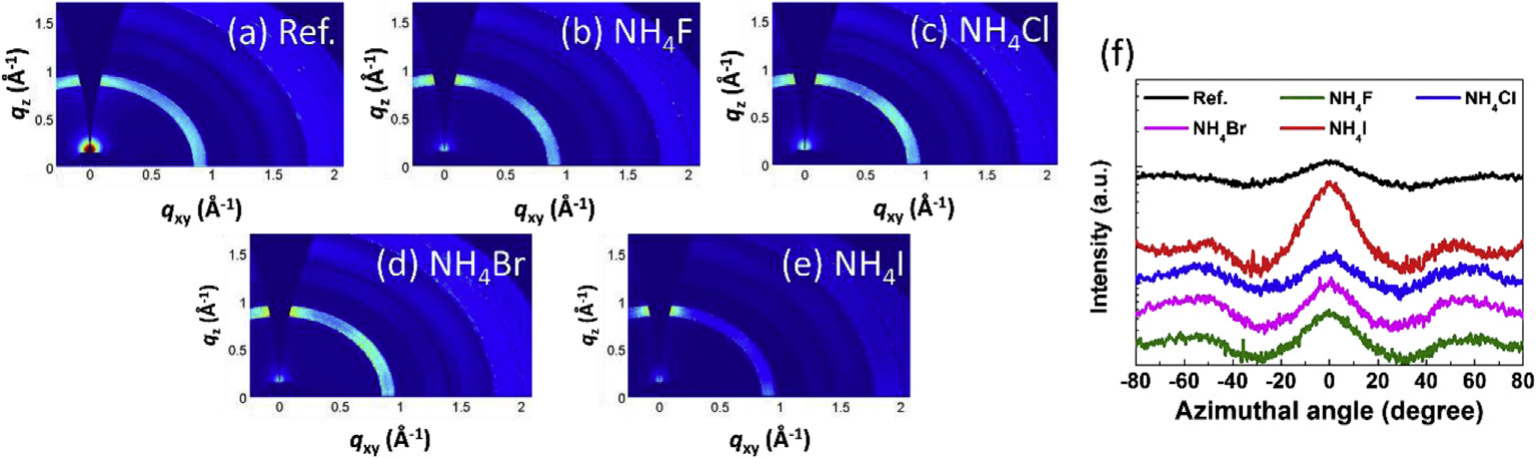
(a–e) 2D images and (f) azimuthal angle scans for (110) peak at around *q* = 0.98 Å^−1^ in the GIWAXS patterns of perovskite films.

**Fig. 3:**
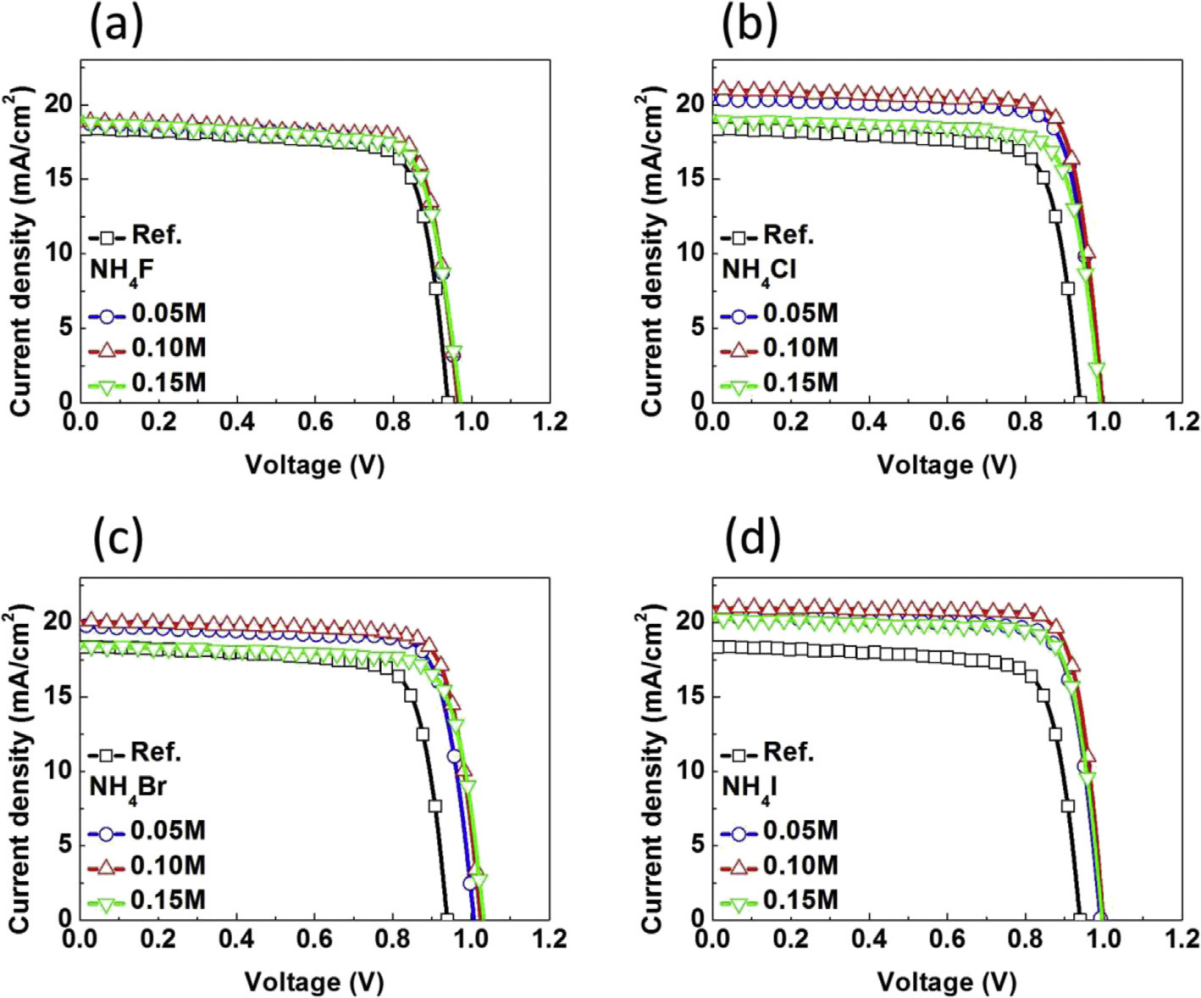
*J-V* characteristics of the reference and NH_4_X (X = F, Cl, Br, I) incorporating MAPbI_3_ inverted planar PvSCs with different amount of ammonium halide additives.

**Fig. 4:**
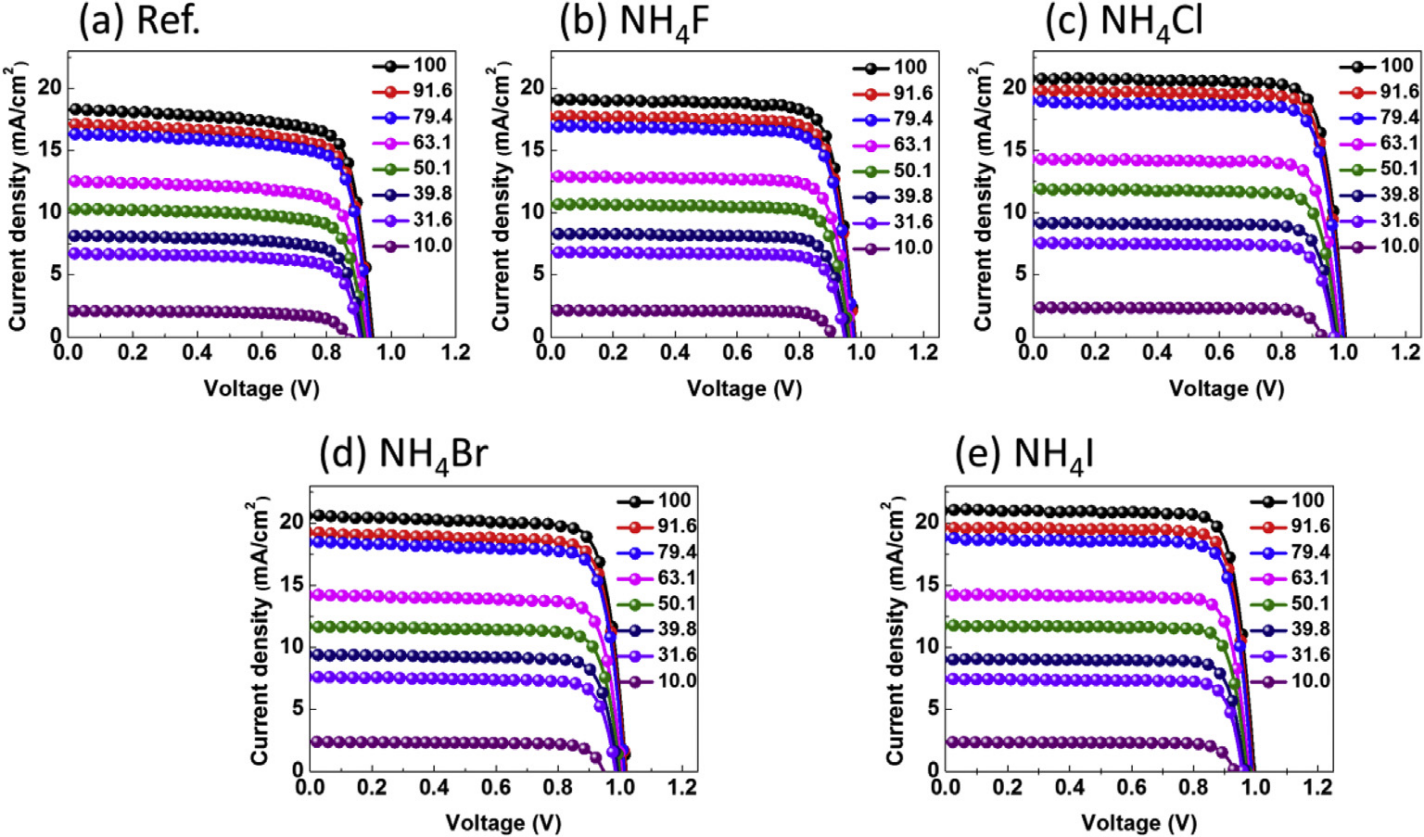
*J-V* characteristics of the (a) reference, and (b–e) NH_4_X (X = F, Cl, Br, I) incorporating MAPbI_3_ inverted planar PvSCs with respect to change in the light intensity.

**Fig. 5:**
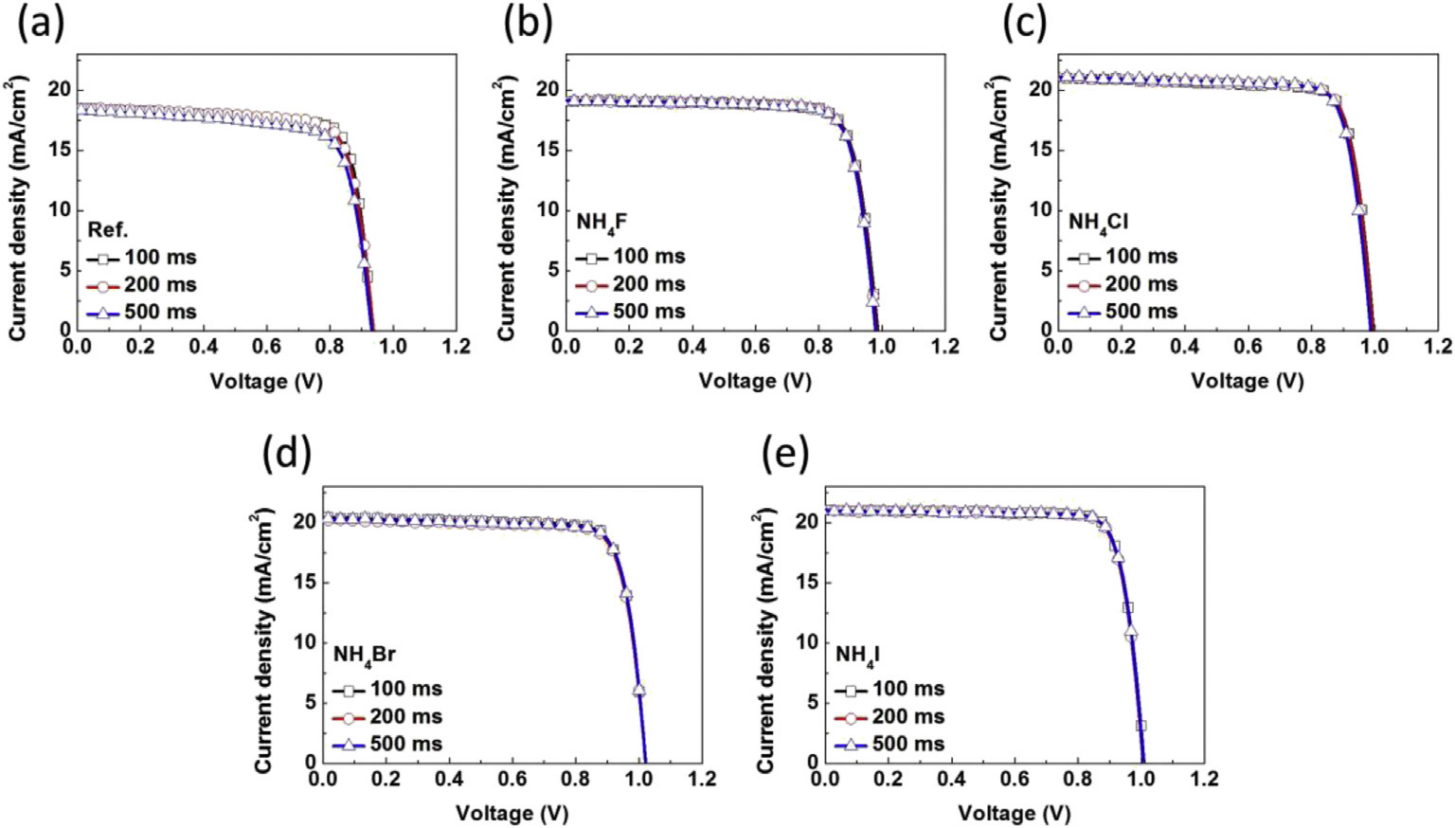
The *J-V* curves of (a) reference and (b–e) NH_4_X (X = F, Cl, Br, I) incorporating MAPbI_3_ PvSCs with different delay time (100–500 ms per 0.01 V) under the reverse scan direction.

**Fig. 6:**
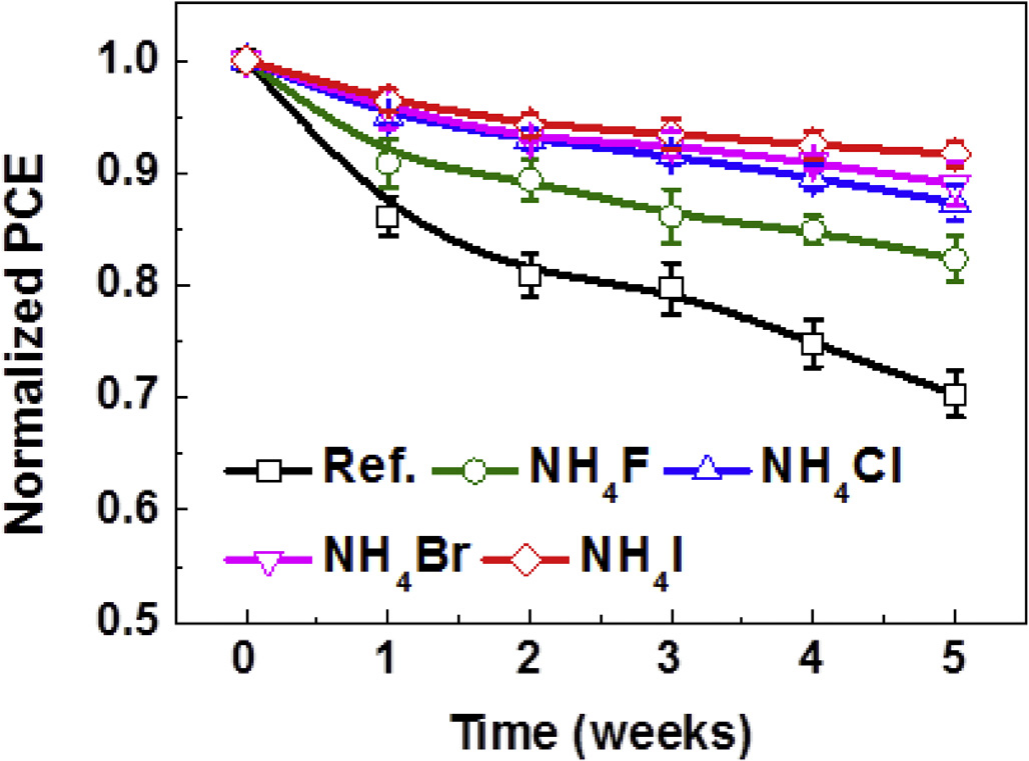
Normalized PCE of reference and NH_4_X (X = F, Cl, Br, I) incorporating MAPbI_3_ inverted planar PvSCs with respect to time.

This data set shows the effect of small amount of the organic cationic material NH_4_X (X = F, Cl, Br, I) on the device PCEs and stability. Fig. 1 describes the nucleation behaviour with the CB dripping time. Fig. 2 describes the crystalline orientation of the perovskite films via GIWAXS. Fig. 3 describes JV curves of different additives incorporated perovskite solar cells. Fig. 4 describes JV characteristics of different additives incorporated perovskite solar cells under different light intensities. Fig. 5 describes JV characteristics of different additives incorporated perovskite solar cells under different bias speed. Fig. 6 displays normalized PCEs of different additives incorporated perovskite solar cells with respect to time of 5 weeks.

## 2 Experimental design, materials, and methods

### 2.1 Materials and preparation of perovskite precursor solution

Lead iodide (99.999% trace metals basis), ammonium fluoride (NH_4_F) (≥99.99% trace metals basis), ammonium chloride (NH_4_Cl) (99.99% trace metals basis), ammonium bromide (NH_4_Br) (99.999% trace metals basis), ammonium iodide (NH_4_I) (99.999% trace metals basis), dimethyl sulfoxide (DMSO), ɣ-butyrolactone (GBL) and chlorobenzene (CB) were purchased from Sigma-Aldrich. Methylammonium iodide (MAI) was purchased from Dyesol and all the materials were used as received without any further purification. To prepare the perovskite precursor solution, we mixed MAI (159 mg) powder and PbI_2_ (461 mg) (1:1 M ratio) in 1 mL mixed GBL:DMSO (0.7:0.3) solvent for the reference perovskite precursor solution [[1], [2], [3], [4], [5], [6]]. Whereas, for the NH_4_X (X = F, Cl, Br, I) incorporated MAPbI_3_ perovskite solution, 0.10 M of NH_4_F (3.70 mg), NH_4_Cl (5.34 mg), NH_4_Br (9.79 mg) and NH_4_I (14.49 mg) were added in the reference perovskite precursor solution. All perovskite precursor solutions were kept for stirring at 70 °C for overnight before use. The important point here to be noted is that the solubility of NH_4_F is very low. Although, we added very small amount (3.70 mg) in 1 mL reference perovskite precursor solution but it was not well soluble and need to filter to remove the insoluble NH_4_F. Whereas, other ammonium halide materials show good solubility with given quantities.

### 2.2 Device fabrication

For inverted planar perovskite solar cells device fabrication, firstly, the patterned glass/ITO substrates were cleaned with DI water, acetone and isopropanol and dried in drying oven at 140 °C for overnight. PEDOT:PSS (Clevios P VP AI4083) was spin-coated on UV-ozone treated glass/ITO substrates at 4000 rpm for 60 sec in air and dried at 150 °C for 20 min [[7], [8], [9]]. Then, the samples were transferred to the N_2_ filled glove-box for further device fabrication steps. Perovskite precursor solution without and with NH_4_X (X = F, Cl, Br, I) was spin coated in N_2_ filled glove-box at 2000 rpm for 60–70 sec, followed by a step of 1000 rpm for 20 sec. During the 2nd step of 2000 rpm for 60 sec, a chlorobenzene (CB) solution (400 μL) was dropped on the substrate during spin coating after 40 sec and continued the spin for further 20 sec [[8], [9], [10], [11]]. The important point to be noted here that the CB dripping time during 2nd spin-coating step was further delayed approximately 5–10 sec for NH_4_X (X = F, Cl, Br, I) containing perovskite precursor solutions as compare to reference solution. Then, the samples were dried on hot plate at 100 °C for 3 min. Then PC_61_BM (purchased from OSM, Republic of Korea) as ETL was deposited on the glass/ITO/PEDOT:PSS/perovskite substrate by spin coating PC_61_BM (20 mg/1 mL in CB) solution at 1200 rpm for 30 sec followed by a final spin-coating step of 2000 rpm for 2 sec. Finally, the LiF/Al (0.5nm/100nm) electrode was deposited by thermal evaporation.
